# Supplementary material for: Machine Learning Accelerates Crystallization for Structure Determination
Source: Angew Chem Int Ed Engl. 2026 May 4;65(25):e1218503. doi: 10.1002/anie.1218503 (PMC13266958; doi:10.1002/anie.1218503)

checkCIF/PLATON report

Structure factors have been supplied for datablock(s) lcz092

THIS REPORT IS FOR GUIDANCE ONLY. IF USED AS PART OF A REVIEW PROCEDURE FOR PUBLICATION, IT SHOULD NOT REPLACE THE EXPERTISE OF AN EXPERIENCED CRYSTALLOGRAPHIC REFEREE.

No syntax errors found.   CIF dictionary   Interpreting this report

Datablock: lcz092

Bond precision:	C-C = 0.0247 A		Wavelength=1.54184
Cell:	a=12.2200(2)	b=12.4005(2)	c=33.2147(4)
	alpha=81.750(1)	beta=81.509(1)	gamma=63.477(2)
Temperature:	100 K		

	Calculated	Reported
Volume	4436.95(14)	4436.95(14)
Space group	P -1	P -1
Hall group	-P 1	-P 1
Moiety formula	C39 H19 Ag3 F18 N6 S4	C15 H3 Ag3 F18 N6, 2(C12 H8 S2)
Sum formula	C39 H19 Ag3 F18 N6 S4	C39 H19 Ag3 F18 N6 S4
Mr	1365.45	1365.45
Dx,g cm-3	2.044	2.044
Z	4	4
Mu (mm-1)	13.343	13.343
F000	2648.0	2648.0
F000'	2664.20	
h,k,lmax	14,14,39	14,14,39
Nref	15698	15181
Tmin,Tmax	0.077,0.263	0.455,1.000
Tmin'	0.010	

Correction method= # Reported T Limits: Tmin=0.455 Tmax=1.000
AbsCorr = MULTI-SCAN

Data completeness= 0.967 Theta(max)= 66.599

R(reflections)= 0.0950(14187) wR2(reflections)= 0.2906(15181)

S = 1.116 Npar= 1261

The following ALERTS were generated. Each ALERT has the format
test-name_ALERT_alert-type_alert-level.
Click on the hyperlinks for more details of the test.

Alert level A

PLAT971_ALERT_2_A Check Calcd Resid. Dens. 1.11Ang From Ag03 5.72 eA-3

Author Response: This Alert is due to presence of residual density in the presence of heavy metal atom.

PLAT971_ALERT_2_A Check Calcd Resid. Dens. 1.14Ang From Ag06 5.48 eA-3

Author Response: This Alert is due to presence of residual density in the presence of heavy metal atom.

PLAT971_ALERT_2_A Check Calcd Resid. Dens. 1.18Ang From Ag04 5.10 eA-3

Author Response: This Alert is due to presence of residual density in the presence of heavy metal atom.

PLAT971_ALERT_2_A Check Calcd Resid. Dens. 1.11Ang From Ag05 4.83 eA-3

Author Response: This Alert is due to presence of residual density in the presence of heavy metal atom.

PLAT971_ALERT_2_A Check Calcd Resid. Dens. 0.99Ang From N00W 4.80 eA-3

Author Response: This Alert is due to presence of residual density in the presence of heavy metal atom.

PLAT971_ALERT_2_A Check Calcd Resid. Dens. 0.99Ang From Ag02 4.80 eA-3

Author Response: This Alert is due to presence of residual density in the presence of heavy metal atom.

PLAT971_ALERT_2_A Check Calcd Resid. Dens. 1.86Ang From F00Y 4.48 eA-3

Author Response: This Alert is due to presence of residual density in the presence of heavy metal atom.

PLAT971_ALERT_2_A Check Calcd Resid. Dens. 1.45Ang From F00U 4.40 eA-3

Author Response: This Alert is due to presence of residual density in the presence of heavy metal atom.

PLAT971_ALERT_2_A Check Calcd Resid. Dens. 1.85Ang From C02L 4.24 eA-3

Author Response: This Alert is due to presence of residual density in the presence of heavy metal atom.

PLAT971_ALERT_2_A Check Calcd Resid. Dens. 1.59Ang From F00N 4.22 eA-3

Author Response: This Alert is due to presence of residual density in the presence of heavy metal atom.

PLAT971_ALERT_2_A Check Calcd Resid. Dens. 1.77Ang From F00V 4.12 eA-3

Author Response: This Alert is due to presence of residual density in the presence of heavy metal atom.

PLAT971_ALERT_2_A Check Calcd Resid. Dens. 1.09Ang From Ag03 4.06 eA-3

Author Response: This Alert is due to presence of residual density in the presence of heavy metal atom.

PLAT971_ALERT_2_A Check Calcd Resid. Dens. 1.93Ang From F00Z 3.81 eA-3

Author Response: This Alert is due to presence of residual density in the presence of heavy metal atom.

PLAT971_ALERT_2_A Check Calcd Resid. Dens. 1.04Ang From N017 3.80 eA-3

Author Response: This Alert is due to presence of residual density in the presence of heavy metal atom.

PLAT971_ALERT_2_A Check Calcd Resid. Dens. 1.39Ang From F00I 3.77 eA-3

Author Response: This Alert is due to presence of residual density in the presence of heavy metal atom.

```
PLAT971_ALERT_2_A Check Calcd Resid. Dens.  1.09Ang From Ag06           3.75 eA-3
```

Author Response: This Alert is due to presence of residual density in the presence of heavy metal atom.

```
PLAT971_ALERT_2_A Check Calcd Resid. Dens.  1.57Ang From F00Z           3.56 eA-3
```

Author Response: This Alert is due to presence of residual density in the presence of heavy metal atom.

```
PLAT971_ALERT_2_A Check Calcd Resid. Dens.  1.23Ang From C01P           3.51 eA-3
```

Author Response: This Alert is due to presence of residual density in the presence of heavy metal atom.

Alert level B

```
PLAT097_ALERT_2_B Large Reported Max.  (Positive) Residual Density      5.32 eA-3
```

Author Response: This Alert is due to presence of residual density in the presence of heavy metal atom.

```
PLAT342_ALERT_3_B Low Bond Precision on  C-C Bonds ..............    0.02472 Ang.
```

Author Response: Disordered structure.

```
PLAT971_ALERT_2_B Check Calcd Resid. Dens.  1.01Ang From Ag02           3.40 eA-3
```

Author Response: This Alert is due to presence of residual density in the presence of heavy metal atom.

```
PLAT971_ALERT_2_B Check Calcd Resid. Dens.  1.37Ang From C01Q           3.28 eA-3
```

Author Response: This Alert is due to presence of residual density in the presence of heavy metal atom.

```
PLAT971_ALERT_2_B Check Calcd Resid. Dens.  1.04Ang From Ag04           3.20 eA-3
```

Author Response: This Alert is due to presence of residual density in the presence of heavy metal atom.

```
PLAT971_ALERT_2_B Check Calcd Resid. Dens.  1.15Ang From Ag05          3.08 eA-3
```

Author Response: This Alert is due to presence of residual density in the presence of heavy metal atom.

```
PLAT971_ALERT_2_B Check Calcd Resid. Dens.  1.51Ang From F00R          2.81 eA-3
```

Author Response: This Alert is due to presence of residual density in the presence of heavy metal atom.

```
PLAT972_ALERT_2_B Check Calcd Resid. Dens.  0.92Ang From Ag05         -3.07 eA-3
```

Author Response: This Alert is due to presence of residual density in the presence of heavy metal atom.

```
PLAT972_ALERT_2_B Check Calcd Resid. Dens.  0.87Ang From Ag02         -2.73 eA-3
```

Author Response: This Alert is due to presence of residual density in the presence of heavy metal atom.

```
PLAT972_ALERT_2_B Check Calcd Resid. Dens.  0.84Ang From Ag04         -2.61 eA-3
```

Author Response: This Alert is due to presence of residual density in the presence of heavy metal atom.

Alert level C

```
DIFMX02_ALERT_1_C  The maximum difference density is > 0.1*ZMAX*0.75
            The relevant atom site should be identified.
PLAT029_ALERT_3_C _diffrn_measured_fraction_theta_full value Low .      0.967 Why?
PLAT042_ALERT_1_C Calc. and Reported MoietyFormula Strings  Differ     Please Check
              Calc: C39 H19 Ag3 F18 N6 S4
              Rep.: C15 H3 Ag3 F18 N6, 2(C12 H8 S2)
PLAT084_ALERT_3_C High wR2 Value (i.e. > 0.25) ..................       0.29 Report
PLAT094_ALERT_2_C Ratio of Maximum / Minimum Residual Density ....       2.05 Report
PLAT213_ALERT_2_C Atom F02C            has ADP max/min Ratio .....        3.5 prolat
PLAT213_ALERT_2_C Atom F02H            has ADP max/min Ratio .....        3.5 prolat
PLAT213_ALERT_2_C Atom C01P            has ADP max/min Ratio .....        3.2 prolat
PLAT213_ALERT_2_C Atom F01R            has ADP max/min Ratio .....        3.5 prolat
PLAT220_ALERT_2_C NonSolvent   Resd 1  F   Ueq(max)/Ueq(min) Range        4.3 Ratio
PLAT220_ALERT_2_C NonSolvent   Resd 2  F   Ueq(max)/Ueq(min) Range        3.1 Ratio
PLAT234_ALERT_4_C Large Hirshfeld Difference C01P     --C01Y     .       0.19 Ang.
PLAT234_ALERT_4_C Large Hirshfeld Difference C02E     --C02Z     .       0.19 Ang.
PLAT242_ALERT_2_C Low    'MainMol' Ueq as Compared to Neighbors of       C01U Check
```

```
PLAT242_ALERT_2_C Low    'MainMol' Ueq as Compared to Neighbors of       C028 Check
PLAT906_ALERT_3_C Large K Value in the Analysis of Variance ......      7.419 Check
PLAT906_ALERT_3_C Large K Value in the Analysis of Variance ......      2.249 Check
PLAT911_ALERT_3_C Missing FCF Refl Between Thmin & STh/L=    0.595        515 Report
               -2  3  0, -10  5  0,  -8  5  0,  -9  6  0,  -8  6  0,  -7  6  0,
               -8  7  0,  -7  7  0,  -6  7  0,  -7  8  0,  -6  8  0,  -5  8  0,
               -6  9  0,  -5  9  0,  -4  9  0,  -5 10  0,  -4 10  0,  -3 11  0,
               -2 12  0,   2-12  1,   3-11  1,   4-10  1,   5-10  1,   4 -9  1,
                5 -9  1,   6 -9  1,   5 -8  1,   6 -8  1,   7 -8  1,   6 -7  1,
                   (  485 More Missing: see the .ckf listing file)
PLAT971_ALERT_2_C Check Calcd Resid. Dens.  0.83Ang From C01Q           1.94 eA-3
```

Author Response: This Alert is due to presence of residual density in the presence of heavy metal atom.

```
PLAT971_ALERT_2_C Check Calcd Resid. Dens.  0.71Ang From C01P           1.74 eA-3
```

Author Response: This Alert is due to presence of residual density in the presence of heavy metal atom.

```
PLAT972_ALERT_2_C Check Calcd Resid. Dens.  0.84Ang From Ag06          -2.01 eA-3
```

Author Response: This Alert is due to presence of residual density in the presence of heavy metal atom.

```
PLAT972_ALERT_2_C Check Calcd Resid. Dens.  0.86Ang From Ag03          -2.00 eA-3
```

Author Response: This Alert is due to presence of residual density in the presence of heavy metal atom.

```
PLAT972_ALERT_2_C Check Calcd Resid. Dens.  1.00Ang From Ag03          -1.98 eA-3
```

Author Response: This Alert is due to presence of residual density in the presence of heavy metal atom.

```
PLAT972_ALERT_2_C Check Calcd Resid. Dens.  1.05Ang From Ag01          -1.95 eA-3
```

Author Response: This Alert is due to presence of residual density in the presence of heavy metal atom.

```
PLAT972_ALERT_2_C Check Calcd Resid. Dens.  1.35Ang From Ag01          -1.89 eA-3
```

Author Response: This Alert is due to presence of residual density in the presence of heavy metal atom.

PLAT972_ALERT_2_C Check Calcd Resid. Dens. 0.89Ang From Ag05 -1.84 eA-3

Author Response: This Alert is due to presence of residual density in the presence of heavy metal atom.

PLAT972_ALERT_2_C Check Calcd Resid. Dens. 0.82Ang From Ag01 -1.83 eA-3

Author Response: This Alert is due to presence of residual density in the presence of heavy metal atom.

PLAT972_ALERT_2_C Check Calcd Resid. Dens. 1.05Ang From Ag03 -1.82 eA-3

Author Response: This Alert is due to presence of residual density in the presence of heavy metal atom.

PLAT972_ALERT_2_C Check Calcd Resid. Dens. 1.17Ang From Ag06 -1.81 eA-3

Author Response: This Alert is due to presence of residual density in the presence of heavy metal atom.

PLAT972_ALERT_2_C Check Calcd Resid. Dens. 1.30Ang From Ag06 -1.81 eA-3

Author Response: This Alert is due to presence of residual density in the presence of heavy metal atom.

PLAT972_ALERT_2_C Check Calcd Resid. Dens. 0.89Ang From Ag01 -1.78 eA-3

Author Response: This Alert is due to presence of residual density in the presence of heavy metal atom.

PLAT972_ALERT_2_C Check Calcd Resid. Dens. 1.37Ang From Ag03 -1.77 eA-3

Author Response: This Alert is due to presence of residual density in the presence of heavy metal atom.

PLAT972_ALERT_2_C Check Calcd Resid. Dens. 1.01Ang From Ag04 -1.73 eA-3

Author Response: This Alert is due to presence of residual density in the presence of heavy metal atom.

PLAT972_ALERT_2_C Check Calcd Resid. Dens. 0.97Ang From Ag02 -1.68 eA-3

Author Response: This Alert is due to presence of residual density in the presence of heavy metal atom.

PLAT972_ALERT_2_C Check Calcd Resid. Dens. 1.10Ang From Ag02   -1.68 eA-3

Author Response: This Alert is due to presence of residual density in the presence of heavy metal atom.

PLAT972_ALERT_2_C Check Calcd Resid. Dens. 1.14Ang From Ag06   -1.67 eA-3

Author Response: This Alert is due to presence of residual density in the presence of heavy metal atom.

PLAT972_ALERT_2_C Check Calcd Resid. Dens. 0.95Ang From Ag01   -1.64 eA-3

Author Response: This Alert is due to presence of residual density in the presence of heavy metal atom.

PLAT972_ALERT_2_C Check Calcd Resid. Dens. 1.10Ang From Ag06   -1.62 eA-3

Author Response: This Alert is due to presence of residual density in the presence of heavy metal atom.

PLAT972_ALERT_2_C Check Calcd Resid. Dens. 0.92Ang From Ag02   -1.57 eA-3

Author Response: This Alert is due to presence of residual density in the presence of heavy metal atom.

PLAT972_ALERT_2_C Check Calcd Resid. Dens. 0.88Ang From Ag04   -1.53 eA-3

Author Response: This Alert is due to presence of residual density in the presence of heavy metal atom.

```
PLAT977_ALERT_2_C Check Negative Difference Density on H01O      .      -0.42 eA-3
PLAT977_ALERT_2_C Check Negative Difference Density on H01Q      .      -0.47 eA-3
PLAT977_ALERT_2_C Check Negative Difference Density on H029      .      -0.31 eA-3
PLAT977_ALERT_2_C Check Negative Difference Density on H02A      .      -0.71 eA-3
PLAT977_ALERT_2_C Check Negative Difference Density on H03X      .      -0.78 eA-3
```

Alert level G

```
PLAT003_ALERT_2_G Number of Uiso or U(i,j)  Restrained non-H-Atoms          1 Report
PLAT072_ALERT_2_G SHELXL First  Parameter in WGHT  Unusually Large       0.13 Report
PLAT083_ALERT_2_G SHELXL Second Parameter in WGHT  Unusually Large     231.42 Why ?
PLAT186_ALERT_4_G The CIF-Embedded .res File Contains ISOR Records          1 Report
PLAT242_ALERT_2_G Low    'MainMol' Ueq as Compared to Neighbors of       C03G Check
```

```
PLAT242_ALERT_2_G Low    ’MainMol’ Ueq as Compared to Neighbors of       C03K Check
PLAT242_ALERT_2_G Low    ’MainMol’ Ueq as Compared to Neighbors of       C03P Check
PLAT242_ALERT_2_G Low    ’MainMol’ Ueq as Compared to Neighbors of       C02X Check
PLAT242_ALERT_2_G Low    ’MainMol’ Ueq as Compared to Neighbors of       C037 Check
PLAT242_ALERT_2_G Low    ’MainMol’ Ueq as Compared to Neighbors of       C03J Check
PLAT434_ALERT_2_G Short Inter HL..HL Contact F00G     ..F00Z     .       2.82 Ang.
                                                    -1+x,y,z  =      1_455 Check
PLAT434_ALERT_2_G Short Inter HL..HL Contact F00Z     ..F01G     .       2.82 Ang.
                                                     x,1+y,z  =      1_565 Check
PLAT720_ALERT_4_G Number of Unusual/Non-Standard Labels ..........        178 Note
              Ag01    Ag02    Ag03    Ag04    Ag05    Ag06    S007    S008
              S009    S00A    S00B    S00C    S00D    S00E    F00F    F00G
              F00H    F00I    F00J    F00K    F00L    F00M    F00N    F00O
              F00P    N00Q    F00R    F00S    N00T    F00U    F00V    N00W
              N00X    F00Y    F00Z    N010    F011    F012    F013    F014
              F015    N016    N017    F018    F019    N01A    F01B    F01C
              N01D    F01E    N01F    F01G    F01H    F01I    N01J    F01K
              F01L    F01M    N01N    C01O    H01O    C01P    H01P    C01Q
              H01Q    F01R    C01S    H01S    C01T    C01U    C01V    C01W
              C01X    C01Y    C01Z    H01Z    C020    C021    C022    C023
              H023    C024    C025    C026    C027    H027    C028    C029
              H029    C02A    H02A    C02B    H02B    F02C    C02D    H02D
              C02E    H02E    C02F    H02F    C02G    F02H    C02I    H02I
              C02J    C02K    H02K    C02L    H02L    C02M    C02N    H02N
              C02O    C02P    C02Q    H02Q    C02R    C02S    C02T    H02T
              C02U    C02V    C02W    H02W    C02X    C02Y    C02Z    H02Z
              C030    H030    C031    C032    C033    H033    C034    C035
              C036    H036    C037    C038    C039    C03A    H03A    C03B
              H03B    C03C    C03D    H03D    C03E    H03E    C03F    C03G
              C03H    C03I    H03I    C03J    C03K    C03L    H03L    C03M
              C03N    H03N    C03O    H03O    C03P    C03Q    H03Q    C03R
              H03R    C03S    H03S    C03T    H03T    C03U    H03U    C03V
              C03X    H03X
PLAT860_ALERT_3_G Number of Least-Squares Restraints ............          6 Note
PLAT883_ALERT_1_G Absent Datum for _atom_sites_solution_primary ..     Please Do !
PLAT909_ALERT_3_G Percentage of I>2sig(I) Data at Theta(Max) Still        87% Note
PLAT910_ALERT_3_G Missing FCF Reflection(s) Below Theta(Min)[Deg]=       4.00 Note
                0  0  1,   0  0  2,
PLAT933_ALERT_2_G Number of HKL-OMIT Records in Embedded .res File          7 Note
               -3  0  9,  -2  3  0,  -1  5  6,   0  3 13,   2 -3  4,   3 -4 12,
                4  3 11,
PLAT941_ALERT_3_G Average HKL Measurement Multiplicity ..........         4.9 Low
PLAT969_ALERT_5_G The ’Henn et al.’ R-Factor-gap value ..........      10.415 Note
              Predicted wR2: Based on SigI**2  2.79 or SHELX Weight 26.04
PLAT978_ALERT_2_G Number C-C Bonds with Positive Residual Density.          1 Info
```

```
  18 ALERT level A = Most likely a serious problem - resolve or explain
  10 ALERT level B = A potentially serious problem, consider carefully
  45 ALERT level C = Check. Ensure it is not caused by an omission or oversight
  21 ALERT level G = General information/check it is not something unexpected

   3 ALERT type 1 CIF construction/syntax error, inconsistent or missing data
  76 ALERT type 2 Indicator that the structure model may be wrong or deficient
  10 ALERT type 3 Indicator that the structure quality may be low
   4 ALERT type 4 Improvement, methodology, query or suggestion
   1 ALERT type 5 Informative message, check
```

It is advisable to attempt to resolve as many as possible of the alerts in all categories. Often the minor alerts point to easily fixed oversights, errors and omissions in your CIF or refinement strategy, so attention to these fine details can be worthwhile. It is up to the individual to critically assess their own results and, if necessary, seek expert advice.

Validation response form

Please find below a validation response form (VRF) that can be filled in and pasted into your CIF.

```
# start Validation Reply Form
_vrf_DIFMX02_lcz092
;
PROBLEM: The maximum difference density is > 0.1*ZMAX*0.75
RESPONSE: ...
;
_vrf_PLAT029_lcz092
;
PROBLEM: _diffrn_measured_fraction_theta_full value Low .      0.967 Why?
RESPONSE: ...
;
_vrf_PLAT042_lcz092
;
PROBLEM: Calc. and Reported MoietyFormula Strings  Differ     Please Check
RESPONSE: ...
;
_vrf_PLAT084_lcz092
;
PROBLEM: High wR2 Value (i.e. > 0.25) ..................       0.29 Report
RESPONSE: ...
;
_vrf_PLAT094_lcz092
;
PROBLEM: Ratio of Maximum / Minimum Residual Density ....       2.05 Report
RESPONSE: ...
;
_vrf_PLAT213_lcz092
;
PROBLEM: Atom F02C            has ADP max/min Ratio .....        3.5 prolat
RESPONSE: ...
;
_vrf_PLAT220_lcz092
;
PROBLEM: NonSolvent   Resd 1  F   Ueq(max)/Ueq(min) Range        4.3 Ratio
RESPONSE: ...
;
_vrf_PLAT234_lcz092
;
PROBLEM: Large Hirshfeld Difference C01P     --C01Y     .       0.19 Ang.
RESPONSE: ...
;
_vrf_PLAT242_lcz092
;
PROBLEM: Low    'MainMol' Ueq as Compared to Neighbors of       C01U Check
```

```
RESPONSE: ...
;
_vrf_PLAT906_lcz092
;
PROBLEM: Large K Value in the Analysis of Variance ......      7.419 Check
RESPONSE: ...
;
_vrf_PLAT911_lcz092
;
PROBLEM: Missing FCF Refl Between Thmin & STh/L=    0.595        515 Report
RESPONSE: ...
;
_vrf_PLAT977_lcz092
;
PROBLEM: Check Negative Difference Density on H01O      .      -0.42 eA-3
RESPONSE: ...
;
# end Validation Reply Form
```

PLATON version of 17/09/2025; check.def file version of 17/09/2025

---

duplicate check

No duplication found

Datablock lcz092 - ellipsoid plot

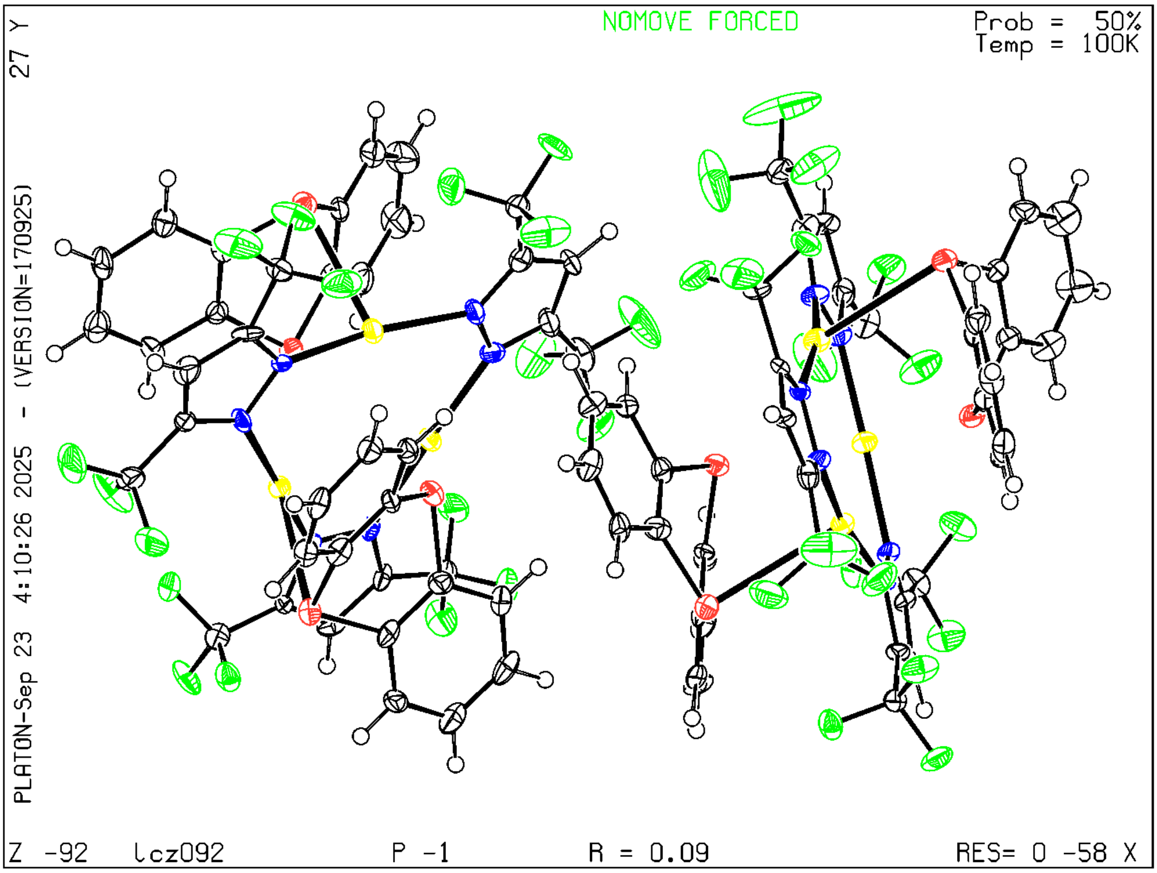

Supplement: Supplementary file 2 — Supporting File 2: anie72462‐sup‐0002‐DataFile.zip. [file ANIE-65-e1218503-s002.zip › supplementary_materials_crystallographic_data submit.zip!/cif_and_checkcif(1-114)/Ag3Pz3_92/Ag3Pz3_92_checkcif.pdf]
